# 6-week radiographs unsuitable for diagnosis of suspected scaphoid fractures

**DOI:** 10.1007/s00402-016-2438-4

**Published:** 2016-03-30

**Authors:** Wouter H. Mallee, Jos J. Mellema, Thierry G. Guitton, J. Carel Goslings, David Ring, Job N. Doornberg, Science of Variation Group

**Affiliations:** 1 Department of Orthopedic Surgery, Academic Medical Center Amsterdam, AMC, Meibergdreef 9, 1105 AZ Amsterdam, The Netherlands; 2 Hand and Upper Extremity Service, Massachusetts General Hospital, Boston, MA USA; 3 Department of Plastic Surgery, University Medical Center Groningen, Groningen, The Netherlands; 4 Department of Trauma Surgery, Academic Medical Center Amsterdam, Amsterdam, The Netherlands; 5 Department of Surgery and Perioperative Care, Dell Medical School, Austin, USA

**Keywords:** Diagnostics, Fracture, Radiographs, Reference standard, Scaphoid, Occult

## Abstract

**Introduction:**

Six week follow-up radiographs are a common reference standard for the diagnosis of suspected scaphoid fractures. The main purpose of this study was to evaluate the interobserver reliability and diagnostic performance characteristics of 6-weeks radiographs for the detection of scaphoid fractures. In addition, two online techniques for evaluating radiographs were compared.

**Materials and methods:**

A total of 81 orthopedic surgeons affiliated with the Science of Variation Group assessed initial and 6-week scaphoid-specific radiographs of a consecutive series of 34 patients with suspected scaphoid fractures. They were randomized in two groups for evaluation, one used a standard website showing JPEG files and one a more sophisticated image viewer (DICOM). The goal was to identify the presence or absence of a (consolidated) scaphoid fracture. Interobserver reliability was calculated using the multirater kappa measure. Diagnostic performance characteristics were calculated according to standard formulas with CT and MRI upon presentation in the emergency department as reference standards.

**Results:**

The interobserver agreement of 6-week radiographs for the diagnosis of scaphoid fractures was slight for both JPEG and DICOM (*k* = 0.15 and *k* = 0.14, respectively). The sensitivity (range 42–79 %) and negative predictive value (range 79–94 %) were significantly higher using a DICOM viewer compared to JPEG images. There were no differences in specificity (range 53–59 %), accuracy (range 53–58 %), and positive predictive value (range 14–26 %) between the groups.

**Conclusions:**

Due to low agreement between observers for the recognition of scaphoid fractures and poor diagnostic performance, 6-week radiographs are not adequate for evaluating suspected scaphoid fractures. The online evaluation of radiographs using a DICOM viewer seem to improve diagnostic performance characteristics compared to static JPEG images and future reliability and diagnostic studies should account for variation due to the method of delivering medical images.

**Level of evidence:**

Diagnostic level II.

## Introduction

In management of suspected scaphoid fractures, overtreatment (i.e. immobilization and restrictions of activities) must be balanced against the risks of nonunion associated with undertreatment [1]. Overtreatment can be limited by establishing early definitive diagnosis using bone scintigraphy [2–4], magnetic resonance imaging (MRI) [5–8] and computed tomography (CT) [5, 8, 9]. However, there is no consensus on scaphoid imaging protocols due to limited evidence regarding diagnostic performance of these advanced imaging techniques [10].

The absence of a consensus reference standard for the diagnosis of scaphoid fractures makes the interpretation of diagnostic performance characteristics and improvement of diagnostic imaging tests difficult [11]. Latent class analysis can be used to estimate diagnostic test accuracy without using a reference standard [1, 12], but this approach has considerable limitations and must be viewed with skepticism [13]. The most commonly used reference standard in studies that evaluated diagnostic tests for scaphoid fractures are scaphoid-specific radiographs made 6 weeks after initial injury [5, 8, 9, 11, 14–18], while some authors question the use of follow-up radiographs as reference standard [19–21].

The Science of Variation Group, a collaborative effort to improve the study of variation in interpretation and classification of injuries, performed numerous studies by evaluating images using JPEG format [22–24]. Since this could limit diagnostic performance due to lack of several functions (window level, zoom, lower quality image), a new online tool was created using an embedded DICOM viewer. This tool mimics clinical practice, however, larger data files and use of multiple functions increases duration of assessment. It is unknown if this tool could be of true value.

As the reliability and accuracy of 6-week radiographs for suspected scaphoid fractures remain subject of discussion and important for the interpretation of diagnostic accuracy of alternative imaging modalities, CT and MRI in particular, there is a need to assess its reliability as well as diagnostic performance characteristics. Therefore, the purpose of this study was to evaluate the interobserver reliability and diagnostic performance characteristics of 6-week radiographs for the recognition of scaphoid fractures in patients with suspected scaphoid fractures. In addition, this study compared the online evaluation of radiographs in JPEG and DICOM format.

## Methods

### Study design

Orthopaedic surgeons affiliated with the Science of Variation Group were asked to log on to http://www.scienceofvariationgroup.org or http://www.traumaplatform.org for an online evaluation of suspected scaphoid fractures. In an invitation email observers were informed that participation would be credited on the study by acknowledgement or group authorship [25, 26] and links were provided that directed to the respective web-based study platforms. Our Institutional Review Board approved this study.

### Subjects

The initial and 6-week radiographs were used from our previous study [5] of a consecutive series of 34 patients aged 18 years or greater with a suspected scaphoid fracture (tenderness of the scaphoid and normal radiographic findings after a fall on the outstretched hand). All patients presented within 24 h after injury and underwent CT and MRI within 10 days after wrist injury between April 2008 and October 2008 in a level I trauma center.

The number of subjects in reliability studies is determined based on an appropriate balance between the number of observers evaluating each subject and the number of subjects [27]. Our web-based study platforms (i.e. Science of Variation Group and Traumaplatform) aim to increase the number of observers in interobserver reliability studies for maximizing power and generalizability and to allow comparison between and within subgroups. For this reason, we prefer to select a limited number of subjects to limit burden on observers and increase participation rate (i.e. number of observers).

### Observers

Orthopedic surgeons trained in hand surgery and listed in the Science of Variation Group as active members were randomized (1:1) by computer-generated random numbers (Microsoft Excel, Redmond, WA, USA) to assess the selected radiographs online in JPEG or DICOM format.

### Online evaluation

Scaphoid-specific radiographs at baseline and 6 weeks after initial trauma were presented and consisted of four views: (1) a posteroanterior view with the wrist in ulnar deviation, (2) a lateral view with the wrist in 15° extension, (3) a lateral view with the wrist in 30° of pronation, and (4) a posteroanterior view with the X-ray beam directed from distal to proximal and with the wrist positioned in 40° of angulation. Observers were asked to answer 1 question for each of the 34 cases: Is there a (consolidated) scaphoid fracture?

Before starting the online evaluation and upon log on to the website, observers received a short description of the study procedure. Observers assigned to the JPEG group evaluated radiographs that were converted to images in JPEG format (http://www.scienceofvariationgroup.org) and observers assigned to the DICOM group evaluated radiographs provided by an online DICOM viewer (http://www.traumaplatform.org). Both groups evaluated the same initial and 6-week radiographs, however, the JPEG group was not able to use the window level, scroll, and zoom options available in the online DICOM viewer software.

### Statistical analysis

A post hoc power analysis was performed using the method as described by Guitton and Ring [23]. It was calculated that 81 observers provided 5.8 % power to detect a 0.003 difference in kappa value (i.e. interobserver reliability) between the JPEG and DICOM group using a two-sample *z* test (alpha = 0.05). However, 81 observers provided 100 % power to detect a clinically relevant difference in kappa value, defined as a difference of one category as describe by Landis and Koch [28] (Δ_kappa_ = 0.20), between the groups with alpha = 0.05.

Interobserver reliability was calculated using the multirater kappa as described by Siegel and Castellan [29]. The kappa statistic is a frequently used measure of chance-corrected agreement between observers and interpreted according to the guidelines of Landis and Koch [28]: a value of 0.01 to 0.20 indicates slight agreement; 0.21 to 0.40, fair agreement; 0.41 to 0.60, moderate agreement; 0.61 to 0.80, substantial agreement; and 0.81 to 0.99, almost perfect agreement. A two-sample *z* test was used to compare kappa values and *P* values of <0.05 were considered significant. For a better understanding of the underlying data, the proportion of agreement was calculated for each case (in absolute percentages, %) and defined as the proportion of observers agreeing with the most provided answer.

Diagnostic performance characteristics (sensitivity, specificity, accuracy, positive predictive value, and negative predictive values) of 6-week radiographs for the recognition of (consolidated) scaphoid fractures were calculated according to standard formulas. The reference standard for the diagnosis of scaphoid fractures was CT and MRI. A panel of three observers, an attending musculoskeletal radiologist, an attending trauma surgeon who treats fractures, and an attending orthopaedic surgeon, evaluated the images for the presence of a scaphoid fracture until a consensus opinion was reached [5]. The 95 % confidence intervals (95 % CIs) were calculated using the formula for the standard error of proportion, based on normal approximation method for binomial proportions, and differences were considered significant when the 95 % CIs did not overlap [30].

## Results

### Observer characteristics

A total of 288 invitation emails were sent, of which 143 went to the JPEG group and 145 to the DICOM group. Fifty-seven respondents started with the evaluation in the JPEG group, of which 53 (93 %) completed the online evaluation, and 45 respondents started in the DICOM group, of which 28 (62 %) completed the online evaluation. After incomplete responses were excluded, 53 (65 %) observers were left in the JPEG group and 28 (35 %) in the DICOM group. Observers were predominately male (95 %), from the US (78 %), hand and wrist surgeons (96 %), and in independent practice for more than 5 years (68 %) (Table 1).

**Table 1:** Observer characteristics

	JPEG (*n* = 53)		DICOM viewer (*n* = 28)	
	*n*	%	*n*	%
Sex				
Men	50	94	27	96
Women	3	5.7	1	3.6
Area				
United States	41	77	22	79
Europe	7	13	3	11
Other	5	9.4	3	11
Specialization				
Hand and wrist	53	100	25	89
Schoulder and elbow	–	–	2	7.1
Trauma	–	–	1	3.6
Years in independent practice				
0–5	18	34	8	29
6–10	9	17	4	14
11–20	16	30	10	36
21–30	10	19	6	21
Fractures per year				
0–10	12	23	5	18
11–20	33	62	7	25
More than 20	8	15	16	57

### Reliability of 6-week radiographs for scaphoid fractures

The interobserver reliability of 6-week radiographs for the diagnosis of scaphoid fractures was the same for the JPEG and DICOM viewer group and slight in both groups (*k* = 0.15 and *k* = 0.14, respectively; *P* = 0.75). In addition, subgroup analysis showed that interobserver agreement ranged from slight to fair and no significant differences in kappa value between subgroups were detected (Table 2). The average proportion of agreement was 68 % in the JPEG group and 68 % in the DICOM group (Table 3).

**Table 2:** Interobserver agreement for the recognition of (consolidated) scaphoid fractures based on 6-week radiographs (JPEG versus DICOM viewer)

	JPEG (*n* = 53)			DICOM viewer (*n* = 28)			*P* value
	Kappa	Agreement	95 % CI	Kappa	Agreement	95 % CI	
Overall	0.15	Slight	0.13 to 0.16	0.14	Slight	0.12 to 0.16	0.75
Area							
United States	0.14	Slight	0.12 to 0.16	0.16	Slight	0.14 to 0.18	0.24
Europe	0.18	Slight	0.09 to 0.26	0.28	Fair	0.06 to 0.50	0.40
Other	0.04	Slight	−0.06 to 0.15	0.18	Slight	−0.04 to 0.41	0.28
Years in independent practice							
0–5	0.12	Slight	0.09 to 0.15	0.06	Slight	0.00 to 0.13	0.094
More than 5 years	0.16	Slight	0.13 to 0.18	0.16	Slight	0.13 to 0.18	0.94
Fractures per year							
0–10	0.17	Slight	0.11 to 0.23	0.22	Fair	0.09 to 0.35	0.47
11–20	0.14	Slight	0.12 to 0.15	0.21	Fair	0.12 to 0.29	0.12
More than 20	0.12	Slight	0.03 to 0.22	0.13	Slight	0.10 to 0.16	0.94

**Table 3:** Proportion of agreement for the recognition of (consolidated) scaphoid fractures based on 6-week radiographs (JPEG and DICOM viewer)

Case no.	JPEG (*n* = 53)		DICOM Viewer (*n* = 28)	
	Most provided answer	PA*	Most provided answer	PA*
1	Present	79	Absent	57
2	Absent	64	Present	86
3	Absent	66	Absent	57
4	Present	57	Absent	75
5	Absent	62	Absent	61
6	Absent	70	Absent	75
7	Absent	57	Present	82
8	Present	51	Absent	75
9	Present	85	Present	57
10	Present	68	Absent	57
11	Present	74	Absent	54
12	Present	72	Present	93
13	Absent	60	Absent	64
14	Absent	83	Absent	71
15	Absent	85	Absent	79
16	Present	62	Present	68
17	Absent	70	Present	86
18	Absent	77	Present	79
19	Absent	55	Present/absent	50
20	Present	53	Absent	61
21	Absent	77	Absent	64
22	Absent	87	Present	61
23	Absent	66	Present	57
24	Present	55	Present	61
25	Absent	74	Present	75
26	Present	70	Absent	64
27	Absent	87	Absent	57
28	Present	62	Absent	61
29	Absent	66	Absent	79
30	Absent	57	Present	75
31	Present	60	Absent	79
32	Absent	87	Absent	71
33	Absent	62	Absent	61
34	Absent	60	Present	61

* Proportion of agreement: the proportion of observers agreeing with the most provided answer

### Diagnostic performance characteristics of 6-week radiographs for scaphoid fractures

The sensitivity of 6-week radiographs for the diagnosis of scaphoid fractures ranged from 42 to 79 % and was significantly higher in the DICOM group compared to the JPEG group with MRI, CT, and MRI with CT combined as reference standard. Specificity ranged from 53 to 59 %, accuracy ranged from 53 to 58 %, and positive predictive value ranged from 14 to 26 % and were not significantly different between the DICOM and JPEG group with MRI, CT and MRI with CT combined as reference standard. The negative predictive value ranged from 79 to 94 % and was significantly higher using the DICOM viewer compared to JPEG images with MRI, CT, and MRI with CT combined as reference standard (Table 4).

**Table 4:** Diagnostic performance of 6-week radiographs for the recognition of (consolidated) scaphoid fractures (JPEG versus DICOM viewer)

	JPEG (*n* = 53)		DICOM viewer (*n* = 28)	
	%	95 % CI	%	95 % CI
Reference standard: MRI				
Sensitivity	42	37–47	64	57–71
Specificity	56	54–59	53	50–57
Accuracy	53	51–56	56	52–59
Positive predictive value	20	17–23	26	22–30
Negative predictive value	79	76–81	85	82–88
Reference standard: CT				
Sensitivity	56	50–62	79	72–85
Specificity	59	56–61	55	51–58
Accuracy	58	56–61	58	55–61
Positive predictive value	19	16–22	23	19–27
Negative predictive value	89	87–90	94	91–96
Reference standard: MRI + CT				
Sensitivity	52	45–59	75	67–83
Specificity	58	55–60	53	50–56
Accuracy	57	55–59	56	52–59
Positive predictive value	14	12–17	18	14–21
Negative predictive value	90	88–92	94	92–96

## Discussion

Scaphoid-specific radiographs at 6 weeks follow-up are most commonly used as reference standard for scaphoid fractures despite its alternatives, such as latent class analysis and MRI, but its use remains subject of discussion [1, 5, 7–9, 12, 14–18]. This study was designed to evaluate the interobserver reliability and diagnostic performance characteristics of 6-week radiographs for the recognition of scaphoid fractures in patients with suspected scaphoid fractures and to compare the online evaluation of radiographs using images in JPEG and DICOM format. We found that the interobserver reliability for 6-week radiographs was slight in both the JPEG and DICOM group. The diagnostic performance characteristics of 6-week radiographs were poor as well, but significantly better when radiographs were evaluated using a DICOM viewer compared to JPEG images.

The strengths of our study include the large number of observers, which allowed a more complex study design with randomization and subgroup analysis, the use of prospectively collected data from our previous study [5] that evaluated a consecutive series of 34 patients with a suspected scaphoid fracture that returned for follow-up after 6 weeks and underwent CT and MRI scans, and the use of DICOM viewers for the online evaluation of radiographs that resembles evaluation in clinical practice. The limitations include the heterogeneous group of surgeons that evaluated the radiographs, which were from multiple countries and different levels of experience and therefore more likely to disagree compared to observers from a single institute with the same level of experience. A possible limitation was the use of a reference standard for the diagnosis of scaphoid fractures that was based on CT and MRI findings and the consensus agreement of three senior authors.

In this study, the interobserver reliability for the recognition of scaphoid fractures based on 6-week radiographs was low in the JPEG and DICOM group and comparable with agreement reported in previous studies [19–21]. Tiel-van Buul et al. [19] selected follow-up radiographs (2 and 6 weeks after injury) of a consecutive series of 60 patients with suspected scaphoid fractures that were rated by 4 observers and found slight to fair interobserver agreement (range *k* = 0.20 to *k* = 0.39). A similar study by Tiel-van Buul et al. [20] reported slight to moderate agreement (range *k* = 0.19 to *k* = 0.50) among 3 observers that evaluated 6-week radiographs of a consecutive series of 78 patients with clinically suspected scaphoid fractures. Low et al. [21] found fair agreement (range *k* = 0.30 to *k* = 0.40) for scaphoid-specific follow-up radiographs between 4 observers that rated 50 patients with a suspected scaphoid fracture.

We found that the diagnostic performance characteristics of 6-week radiographs for scaphoid fractures were poor with MRI, CT, and MRI with CT combined as reference standard using radiographs in JPEG and DICOM format. Six-week radiographs seem better at excluding scaphoid fractures (negative predictive value ranged from 79 to 94 %) than recognizing a scaphoid fracture (positive predictive value ranged from 14 to 26 %). Moreover, our data suggest that almost 50 % of the ratings were inaccurate (accuracy ranged from 53 to 58 %). Low et al. [21]. reported low negative predictive value (range 30 to 40 %) and high positive predictive value (range 75 to 88 %) of follow-up radiographs in patients with suspected scaphoid fractures with MRI as reference standard, which were not consistent with our findings. These differences can be explained as the prevalence influences the negative predictive value and positive predictive value [9, 31]. Our study evaluated the radiographs of a consecutive series of patients (prevalence 18 %) and Low et al. selected patients retrospectively if they had both follow-up radiographs and MRI after injury (prevalence 75 %).

Our results show that the method of presenting radiographs may affect their evaluation by surgeon observers. We found that the interobserver reliability was the same in the JPEG and DICOM group, but the diagnostic performance was better when radiographs were evaluated using a DICOM viewer compared to static JPEG images. The ability to window level, scroll, and zoom using a DICOM viewer improved the diagnosis of scaphoid fractures, in terms of sensitivity and negative predictive value, significantly. Since the format of medical images could be a source of variation between surgeons, it should be accounted for in future reliability and diagnostic studies.

Given the low agreement and poor diagnostic accuracy of 6-week radiographs for the recognition of scaphoid fractures in this study, surgeons and patients must accept that they are dealing with probabilities rather than certainties in the management of scaphoid fractures. For example, we cannot reduce the probability of missing a fracture to 0 % with a negative predictive value of less than 100 %. Using 6-week radiographs as reference standard for studying suspected scaphoid fractures is not advised for future studies. To date, observer experience, training, image presentation, training, and simplification of classifications are shown to have a limited effect on the reliability and accuracy of diagnosis and classification of fractures. At this time it remains unclear what interventions will improve reliability and accuracy, but our collaborative plans to continue studying variation between surgeons to attempt to reduce it.
